# Peer Victimization among Classmates—Associations with Students’ Internalizing Problems, Self-Esteem, and Life Satisfaction

**DOI:** 10.3390/ijerph14101218

**Published:** 2017-10-13

**Authors:** Sara B. Låftman, Bitte Modin

**Affiliations:** Centre for Health Equity Studies (CHESS), Stockholm University/Karolinska Institutet, SE-10691 Stockholm, Sweden; bitte.modin@chess.su.se

**Keywords:** bullying, victimization, contextual, school climate, well-being, adolescents

## Abstract

Bullying is a major problem in schools and a large number of studies have demonstrated that victims have a high excess risk of poor mental health. It may however also affect those who are not directly victimized by peers. The present study investigates whether peer victimization among classmates is linked to internalizing problems, self-esteem, and life satisfaction at the individual level, when the student’s own victimization has been taken into account. The data were derived from the first wave of the Swedish part of Youth in Europe Study (YES!), including information on 4319 students in grade 8 (14–15 years of age) distributed across 242 classes. Results from multilevel analyses show a significant association between classes with a high proportion of students being victimized and higher levels of internalizing problems, lower self-esteem, and lower life satisfaction at the student level. This association holds when the student’s own victimization has been taken into account. This suggests that peer victimization negatively affects those who are directly exposed, as well as their classmates. We conclude that efficient methods and interventions to reduce bullying in school are likely to benefit not only those who are victimized, but all students.

## 1. Introduction

Bullying and peer victimization in the school setting is a problem that often has severe consequences for the exposed student’s mental health [1,2,3,4] and wellbeing [5,6]. In Sweden, the proportion of students who are subjected to bullying at least 2–3 times a month has been estimated to be around 3–6%, with a further 10% experiencing peer victimization less frequently [7,8]. The concept of ‘bullying’ [9] holds special criteria when it comes to operationalization and measurement compared to the broader and less distinct term of ‘peer victimization’. It is arguably important to make a conceptual distinction between persistent and systematic exposure to bullying and more temporary and transient episodes of victimization—not least out of concern for the individual students in question. In this study, we use the term ‘peer victimization’ to underscore the fact that our measure does not make a strict distinction between those who are systematically bullied and those who are occasionally victimized by peers. However, since our main question concerns ‘concentrations’ of peer victimization at the class level rather than individual victimization, a measure that captures as many manifestations of peer victimization as possible is preferable to a more restricted one [10].

When an individual student develops psychological problems because he or she is victimized, it can be seen as an individual-level effect: the victimization has consequences for the mental well-being of the individual who is directly exposed. However, the existence of peer victimization in the school setting can also have consequences for the overall school climate, and thus ‘spill over’ onto the mental well-being of the surrounding peer group. Students who participate in, witness, or are simply aware of such bullying behaviors in their class are likely to sense that the social climate is unfriendly and may also become worried that they, for example, might be the next victim. The ‘concentration’ of peer victimization in a class may, in this way, lead to poorer mental well-being among classmates who are not directly exposed. Further, the more common peer victimization is, the higher is the risk that students who are not victimized themselves will become aware of or witness such situations and be affected. The mere fact that peer victimization is known of or observed in the immediate environment can thus be seen as a marker of “ambient threat” in the school setting [11] (p. 386).

Accordingly, in classes where peer victimization is common, students who are not yet victimized may perceive a potential risk of becoming a future target. This may lead to elevated levels of stress, a more negative self-perception, and poorer general well-being. However, to the best of our knowledge, only a small number of empirical studies have hitherto investigated contextual effects of bullying among classmates and students’ mental well-being, i.e., whether the occurrence of bullying in a class is linked to students’ mental well-being even when adjusting for peer victimization at the student level. Using data from the Stockholm School Survey of students in grade 9 (15–16 years of age), Modin and Östberg [12] found that the proportion of students in a class who reported being subjected to harassment was associated with poorer individual psychosomatic health, even when the student’s own exposure to harassment had been adjusted for. A similar pattern was shown for the prevalence of bullying in the class and students’ psychosomatic health, although this association was seen only among girls [11]. In a study based on the Danish part of the Health Behavior of School-aged Children (HBSC) survey conducted among students aged 11, 13, and 15, Meilstrup et al. [13] demonstrated a link between higher numbers of students in a class reporting being bullied and higher levels of reported emotional symptoms, even when victimization at the individual level had been controlled for. These findings can be seen as indicating that bullying and peer victimization have negative effects on mental well-being, not only among students who are directly involved, but for the class as a whole. Even though it seems reasonable to assume a causal relationship between the proportion of victimized students in a class and students’ mental well-being, it should be acknowledged that the association may be bidirectional. It is possible that a high concentration of students with poor mental well-being entails a higher risk of peer victimization occurring. Another possibility is that there are other school-contextual factors—including both organizational characteristics and aspects of the social school climate—which influence both the occurrence of peer victimization and students’ mental well-being. The current study adds to earlier research by investigating associations between the prevalence of peer victimization among classmates and a broader set of measures of well-being—internalizing problems, self-esteem, and life satisfaction—whilst also taking into account peer victimization at the student level.

The purpose of the present study is to analyze whether the prevalence of peer victimization at the class level is related to students’ internalizing problems, self-esteem, and life satisfaction when peer victimization at the student level has been taken into consideration. To this end, we will employ survey data material from grade 8 students (aged 14–15 years) in schools in Sweden. In grade 8 in Swedish schools, classes constitute relatively well-defined units and the students in a class attend most lessons together. Our hypotheses are:

**Hypothesis** **1 (H1).** *A high occurrence of peer victimization at the class level is positively associated with students’ internalizing problems, over and above the individual-level association*.

**Hypothesis** **2 (H2).** A high occurrence of peer victimization at the class level is associated with lower self-esteem among students, over and above the individual-level association.

**Hypothesis** **3 (H3).** A high occurrence of peer victimization at the class level is associated with lower life satisfaction among students, over and above the individual-level association.

## 2. Materials and Methods

The data were derived from the Swedish part of the Youth in Europe Study (YES!), which is part of the larger Children of Immigrants Longitudinal Survey in Four European Countries (CILS4EU) project. The sample was school-based and a stratified sampling strategy was applied. Schools in the sampling frame were assigned to four different strata according to the proportion of students with immigrant background: 0–9%, 10–29%, 30–59%, and 60–100%. First, schools were selected, and thereafter two classes in each school. Four waves have been collected so far. In the current study, data from wave 1 was used. These data were collected in 2010/2011 by means of questionnaires for students in grade 8 (approximately 14–15 years of age) (*n* = 5025 distributed over 251 classes in 129 schools). Additional information about students’ household characteristics was collected through parental questionnaires and register data. More information about the sampling procedure, the data collection and the data material is available elsewhere (www.cils4.eu) [14,15].

### 2.1. Study Sample

Because the study focuses on conditions at class-level, classes with fewer than 10 responding students were omitted to ensure a robust aggregate measure of harassment (71 students). In addition, we omitted cases with missing values for any of the variables included in the analyses (635 students), resulting in a study sample of 4319 students distributed across 242 classes, i.e., 86% of the original sample of 5025 students.

### 2.2. Measures

#### 2.2.1. Dependent Variables

Internalizing problems was constructed from six items about the frequency of feeling worried, anxious or depressed; having had a headache or stomach ache; or having had problems getting to sleep in the previous six months. The first three items were assessed by the question “How often are each of these statements true about you?”, followed by “I feel very worried”, “I feel anxious”, and “I feel depressed”. The response categories were “Often true”, “Sometimes true”, “Rarely true” and “Never true” (coded 3–0). The last three items were assessed by the question “During the past six months, how often have you had…” and the sub-questions “a headache”, “a stomach ache”, and “problems getting to sleep”. The response categories were “Every day”, “Once or several times a week”, “Once or several times a month”, “Less often”, and “Never” (coded 3–0; the two last response categories collapsed). The values of the items were added to form an index ranging between 0 and 18, with higher values indicating more frequent symptoms. The scale shows good internal consistency (Cronbach’s alpha = 0.78). The distribution was skewed towards lower values, meaning that few symptoms were more common and that many symptoms were relatively uncommon. The index was subsequently z-standardized (mean value = 0; standard deviation = 1). The measure has been used in previous studies [16,17,18].

Self-esteem was assessed from the question “How much do you agree or disagree with each of the [following] statements?”: “I have a lot of good qualities”, “I have a lot to be proud of”, and “I like myself just the way I am”. The response categories were “Strongly agree”, “Agree”, “Neither agree nor disagree”, “Disagree”, and “Strongly disagree”. The five-point scale was coded so that for each item, “Strongly agree” was given the value of 4 and “Strongly disagree” the value of 0. Since the three items were correlated (Cronbach’s alpha = 0.84) they were added to a form an index with the range 0–12. The distribution was heavily skewed towards higher values in that more students tended to have high self-esteem. Accordingly, we constructed an ordinal scale measure with three categories of about equal size (values 0–8; 9–10; and 11–12, respectively).

Life satisfaction was captured by the question “On a scale of 1 to 10, where 1 is very unsatisfied and 10 is very satisfied, how satisfied are you with your life in general?” The distribution of this measure was heavily skewed towards higher values since more students tended to rate their life satisfaction as high. We constructed an ordinal scale measure with three categories of as similar size as possible (values 1–7; 8–9; and 10, respectively).

#### 2.2.2. Independent Variables

Peer victimization was measured by the question “How often have the following things happened in the last month?” followed by the statements: “I was scared of other students”, “I was teased by other students”, and “I was bullied by other students”. The response alternatives were: “Every day”, “Once or several times a week”, “Less often”, and “Never” (coded 3–0). The three items have a good internal consistency (Cronbach’s alpha = 0.74). Responses from the three items were added to form an index ranging from 0 to 9, with higher values indicating greater exposure to harassment. Students with values >1 standard deviation above the mean were classified as being subjected to harassment. This strategy was adopted from earlier studies that used the KIDSCREEN social acceptance subscale which is based on items similar to the ones used here [19,20].

Peer victimization at the class level was calculated as the proportion of students in each class who were classified as being victimized by peers (measured in %). To detect potential non-linear associations, the continuous measure was subsequently divided into three categories of about equal size, in order to distinguish classes with a relatively low, intermediate, and high occurrence of peer victimization.

#### 2.2.3. Control Variables

Sex was based on information from the student questionnaire, with the categories boy and girl, respectively.

Age was the year when the student filled in the survey (i.e., 2010 or 2011) minus the student’s birth year.

Family structure was based on information in the student questionnaire about whether or not the student lived with two custodial parents in the same household. This was included as a control variable since earlier research has shown that adolescents not living with two custodial parents in the same have a higher risk of both peer victimization [21] and of poor mental well-being [16,22] compared with those living with two custodial parents in the same household.

Parents’ occupational status was included as a control variable since household socioeconomic conditions have been shown to be linked with a higher risk of peer victimization [23,24] as well as of various sorts of mental health problems [25]. The measure was primarily constructed from parent-reported information and secondarily from student-reported information and coded according to the international classification system ISEI [26,27]. The parent was asked to provide the title and a short description of his/her own and his/her partner’s jobs. If the parent had not completed the questionnaire, information about parents’ occupational status was instead collected from the students’ questionnaire, in which the student was asked to provide the title and a short description of both parents’ jobs. In two-parent families, parents’ occupational status was defined as the highest ISEI score.

Immigrant background was included as a control variable since earlier studies based on the same data material have demonstrated that peer victimization is less commonly experienced by second generation immigrants than among the Swedish majority [21] and that mental well-being is higher among students with immigrant backgrounds compared to the Swedish majority [17,18]. The variable was based on information in the student questionnaire about the parents’ country of birth. Students with at least one parent born in Sweden were classified as not having an immigrant background. Students whose parents (or the only parent) were born in a country other than Sweden were coded as having an immigrant background.

### 2.3. Ethics

The Regional Ethical Review Board of Stockholm has given ethical approval to the project (2010/1557-31/5).

### 2.4. Statistical Analysis

The study uses multilevel analysis, which is appropriate since the data structure is hierarchical, with students nested in classes. The software is Stata version 13.1. We performed two-level linear regression models of internalizing problems (using the ‘xtmixed’ command) and two-level ordered regression models of self-esteem and life satisfaction (using the ‘meologit’ command), respectively. To account for differences in sampling probabilities we used a sampling weight in the descriptive analyses. In the multilevel regression analyses we adjusted for stratum (i.e., dummy variables for the strata used in the sampling of schools).

### 2.5. Analytical Strategy

In the analyses, we first estimated whether exposure to peer victimization was predictive of internalizing problems, self-esteem and life satisfaction after control for sociodemographic background characteristics (student level). These analyses are presented in Models 1. Next, we added class-level proportions of peer victimization to the model to investigate if there was any additional disadvantage for individual students of being part of a class context where peer victimization is common (contextual level). These analyses are presented in Models 2.

## 3. Results

A description of the data is provided in Table 1. It shows that, according to our operationalization, 15.3% of the students were victimized by peers.

Table 2 gives information about the proportion of students who were victimized by peers in each of the three class-level categories of peer victimization. In the classes where peer victimization was least common (‘lowest third’), 4.8% of the students reported to be victimized. In the intermediate third, 13.9% of the students reported to be victimized. In the category where peer victimization was most common (‘highest third’), 26.8% of the students reported to be victimized. Table 2 also presents the mean values of internalizing problems and the percentages of students reporting different levels of self-esteem and life satisfaction, respectively, according to the three categories of class-level peer victimization. For the distribution of internalizing problems, self-esteem, and life satisfaction, we can see clear gradients by the three categories of class-level peer victimization. The mean value of internalizing problems was lowest in classes where peer victimization was relatively uncommon (−0.12) and highest in those where it was relatively common (0.16). Furthermore, both self-esteem and life satisfaction were higher among students attending classes where peer victimization was relatively uncommon, and lower among students in classes where peer victimization was relatively frequent. For instance, among students in the lowest third—i.e., in classes with a relatively low level of peer victimization—34.5% reported high self-esteem. Among students in the highest third—i.e., in classes with a relatively high level of peer victimization—the corresponding proportion was 28.3%. Similarly, for life satisfaction, among students in the lowest third (i.e., classes where peer victimization was relatively uncommon), 27.8% reported high life satisfaction, compared with 20.9% of the students in the highest third (i.e., classes where peer victimization was relatively common).

To assess whether the patterns in Table 2 change in a multiple regression framework where the individual student’s victimization is also taken into consideration, results from multilevel regression models are presented in Table 3. For internalizing problems, we performed two-level linear regression analyses. Due to their skewed distributions, self-esteem and life satisfaction were analyzed by means of two-level ordered regression models. For all outcomes, Model(s) 1 include only individual-level variables. Model(s) 2 add class size and the three categories of class-level harassment. Results show that—whilst controlling for students’ sex, age, family type, parental social class, and immigrant background—students who were victimized by peers reported significantly more internalizing problems (b = 0.73, *p* < 0.001) as well as lower self-esteem (OR = 0.45, *p* < 0.001) and lower life satisfaction (OR = 0.38, *p* < 0.001) than those who were not victimized. Model(s) 2 further demonstrate that the proportion of classmates who were victimized by peers—independently of class size, individual victimization, and the variables adjusted for in Model 1—was significantly associated with all three outcomes in the expected direction. Thus, a relatively high concentration of peer victimization in the class was predictive of more internalizing problems (b = 0.08, *p* < 0.05), lower self-esteem (OR = 0.81, *p* < 0.05) and lower life satisfaction (OR = 0.84, *p* < 0.05) than among students exposed to lower concentrations of peer victimization in the class. For life satisfaction, students in the intermediate category of class-level victimization (OR = 0.81, *p* < 0.05) tended to report lower life satisfaction than students in classes where there was a low level of peer victimization.

As an additional check (not presented), we also performed analyses of the continuous measure of class-level peer victimization, i.e., the proportion of students in a class who reported to be victimized (ranging between 0 and 47%) instead of the three categories. For all three outcomes, the estimate of the continuous measure was statistically significant (for internalizing problems b = 0.01, *p* < 0.01; for self-esteem OR = 0.99, *p* < 0.01; for life satisfaction OR = 0.99, *p* < 0.05) (not presented).

In the ordered multilevel regression models, the parallel lines assumption (i.e., that the distance between each category of the dependent variable is equivalent) was not met. Therefore, to check the robustness of the findings, we also performed binary logistic multilevel models with dichotomous versions of self-esteem and of life satisfaction. For self-esteem, we compared students with low vs. intermediate/high self-esteem, as well as those with low vs. high self-esteem (omitting individuals with intermediate self-esteem from the analysis). Both analyses produced results similar to the ones from the ordered multilevel regression analysis: students in classes in the highest of the three peer victimization categories were less likely to report intermediate/high or high self-esteem than those in the lowest third (data not presented). Similarly, for life satisfaction we compared students with low vs. intermediate/high life satisfaction, as well as those with low vs. high life satisfaction (omitting individuals with intermediate life satisfaction). In both analyses, results were similar to the ones produced in the ordered multilevel regression model (although in the analyses of students with low vs. intermediate/high life satisfaction, the estimate of the highest third of class-level peer victimization did not reach statistical significance (*p* = 0.102), data not presented). Since these additional checks showed very similar results to those displayed in Table 3, we prefer to report the results of the ordered multilevel regression models to make use of as much of the variation in the data as possible.

Furthermore, as an additional check, we performed analyses of peer victimization operationalized in a slightly different way (at both the individual- and the class-level), in accordance with previous studies that have used the same data material [21,24]: a student was classified as victimized by peers if he or she reported to have been bullied, teased and/or afraid of other students at least once a week, or having experienced all three types of events some time during the past month. These analyses, with a slightly lower proportion of students classified as victimized by peers (9.6%), showed similar results to the ones presented albeit with some minor differences in significance levels (data not presented), indicating that the patterns presented in Table 3 seem rather robust.

## 4. Discussion

Using Swedish survey data collected from students in grade 8 (aged 14–15 years), the present study shows that the class-level concentration of peer victimization was linked to higher levels of internalizing problems as well as to lower self-esteem and lower life satisfaction, even when peer victimization at the individual level was adjusted for. Thus, our findings indicate that there are contextual effects of peer victimization among classmates with potentially harmful implications for the class as a whole. The effect sizes, although not as resolute as at individual-level, were not negligible. The strongest associations were found at the individual level, with students who themselves were victimized reporting more internalizing problems, lower self-esteem and lower life satisfaction than their classmates who were not victimized. This corroborates findings from earlier studies [1,2,3,4,5,6].

Where the contextual effects of peer victimization are concerned, the present study confirms and extends previous research. By analyzing three important aspects of well-being—internalizing problems, self-esteem, and life satisfaction—we conclude that the class-level concentration of peer victimization, over and above individual victimization, is negatively linked not only with emotional [13] and psychosomatic symptoms [11,12] as has been shown in earlier studies, but also with mental well-being in a broader sense. One interpretation of our findings is that students who know of or observe victimization among peers experience that the social climate is unfriendly and may also sense that they are potential future targets. This in turn causes higher levels of stress, lower self-perception, and lower overall well-being. For internalizing problems and self-esteem, statistically significant differences were observed only between the lowest vs. highest of the three categories of class-level concentration of peer victimization. For life satisfaction, both an intermediate and a high concentration of peer victimization demonstrated a statistically significant effect, compared with a low concentration. Taken together, our results suggest that classes where peer victimization is relatively rare are positive for mental well-being.

The data were based on a survey conducted among students in schools with high response rates (school level: 77%, class level: 99%, student level: 86%) (CILS4EU 2016, Table 11) [15]. One limitation is the fact that external non-response at the student level is likely to be systematic. For instance, it seems plausible that students with high levels of internalizing problems, low self-esteem, and low life satisfaction were more likely to be absent from school on the day of the survey and therefore are underrepresented in the data. However, we do not see any reason to believe that this potential bias may have influenced our findings to any substantial extent. If anything, it seems likely that the reported associations were under- rather than over-estimated. Another limitation is that, because the data are cross-sectional, it does not enable us to draw any conclusions about causality. Nor can we rule out the possibility that there may be other aspects of the class context, not observed here, that are linked to both peer victimization and students’ mental well-being.

Both the current study and earlier research into the contextual effects of bullying are based on analyses of Scandinavian data [11,12,13]. Since Sweden in particular is recognized as a country with a low prevalence of school bullying [28], it is not easy to generalize these findings to other national contexts. Further investigations using data from other countries would therefore seem to be a promising field for future research.

## 5. Conclusions

The finding that the prevalence of peer victimization in Swedish classes is related to poorer mental well-being not only for those who are victimized themselves but for all students is highly important from a policy perspective. These results help us to understand peer victimization and bullying as group phenomena and how negative emotions and reactions related to the prevalence of peer victimization appear to ‘spread’ among students. Conversely, it is also important to note that students in classes with relatively low concentrations of peer victimization tend to report more positive outcomes. We can therefore conclude that effective measures to combat peer victimization and bullying will benefit all students.

## Figures and Tables

**Table 1:** Descriptives (unweighted), *n* = 4319.

**Dependent Variables**	**%**	***n***		
Self-esteem				
Low	31.1	1341		
Intermediate	33.4	1443		
High	35.5	1535		
Life satisfaction	%	*n*		
Low	28.8	1246		
Intermediate	44.5	1921		
High	26.7	1152		
	Mean	s.d.	Min	Max
Internalizing problems	4.77	3.45	0	18
(std)	0	1	−1.39	3.84
**Independent Variables**	**%**	***n***		
Peer victimization	15.3	659		
	Mean	s.d.	Min	Max
Peer victimization at the class level	0.15	0.10	0	0.47
**Control Variables**	**%**	***n***		
Sex				
Boys	48.3	2088		
Girls	51.7	2231		
Family type				
Two custodial parents	66.8	2885		
Other	33.2	1434		
Immigrant background				
No	70.3	3038		
Yes	29.7	1281		
	Mean	s.d.	Min	Max
Age	14.78	0.50	13	17
Parents’ occupational status	49.89	16.73	16	89
Class size	21.29	4.07	10	32

s.d.: standard deviation; std: standardized.

**Table 2:** Descriptives of the lowest, intermediate, and highest categories of peer victimization at the class level: mean values of internalizing problems, and percentages of low, intermediate, and high self-esteem and life satisfaction, respectively. Weighted data.

Peer Victimization at the Class Level	*n*	Internalizing Problems	Self-Esteem (%)			Life Satisfaction (%)		
		(Mean)	Low	Inter	High	Low	Inter	High
Lowest third (4.8%)	1489	−0.12	28.5	37.0	34.5	25.5	46.7	27.8
Intermediate third (13.9%)	1421	0.00	31.8	36.5	31.7	30.0	48.1	21.9
Highest third (26.8%)	1409	0.16	39.4	32.3	28.3	31.9	47.2	20.9

**Table 3:** Coefficients from two-level linear regression models (internalizing problems) and odds ratios from two-level ordered regression models (self-esteem and life satisfaction). Model(s) 1 include peer victimization at the individual level, adjusted for sex, age, family type, immigrant background, and parental social class. Model(s) 2 add class size and peer victimization at the class level. All models adjusted for stratum (4319 students distributed across 242 classes).

**Internalizing Problems**	**Model 1**	**Model 2**
	**b**	**b**
Peer victimization	0.73 ***	0.72 ***
Peer victimization at the class level		
Lowest third (ref.)		0.00
Intermediate third		0.07
Highest third		0.08 *
Class-level variance (s.d.)	0.14 (0.02)	0.13 (0.02)
**Self-Esteem**	**Model 1**	**Model 2**
	**OR**	**OR**
Peer victimization	0.45 ***	0.47 ***
Peer victimization at the class level		
Lowest third (ref.)		1.00
Intermediate third		0.89
Highest third		0.81 *
Class-level variance (s.d.)	0.09 (0.03)	0.09 (0.03)
**Life Satisfaction**	**Model 1**	**Model 2**
	**OR**	**OR**
Peer victimization	0.38 ***	0.39 ***
Peer victimization at the class level		
Lowest third (ref.)		1.00
Intermediate third		0.81 *
Highest third		0.84 *
Class-level variance (s.d.)	0.08 (0.03)	0.07 (0.03)

*** *p* < 0.001, ** *p* < 0.01, * *p* < 0.05.

## References

[1] Due P., Holstein B.E., Lynch J., Diderichsen F., Nic Gabhain S., Scheidt P., Currie C. (2005). The Health Behavior in School-Aged Children Bullying Working Group. Bullying and symptoms among school-aged children: International comparative cross sectional study in 28 countries. Eur. J. Public Health.

[2] Låftman S.B., Östberg V. (2006). The pros and cons of social relations. An analysis of adolescents’ health complaints. Soc. Sci. Med..

[3] Stassen Berger K. (2007). Update on bullying at school: Science forgotten?. Dev. Rev..

[4] Gini G., Pozzoli T. (2009). Association between bullying and psychosomatic problems: A meta-analysis. Pediatrics.

[5] Tsaousis I. (2016). The relationship of self-esteem to bullying perpetration and peer victimization among schoolchildren and adolescents: A meta-analytic review. Aggress. Violent Behav..

[6] Fantaguzzi C., Allen E., Miners A., Christie D., Opondo C., Sadique Z., Fletcher A., Grieve R., Bonell C., Viner R.M. (2017). Health-related quality of life associated with bullying and aggression: A cross-sectional study in English secondary schools. Eur. J. Health Econ..

[7] Swedish National Agency for Education (2009). På Tal om Mobbning—Och Det som Görs. Kunskapsöversikt.

[8] Swedish National Institute of Public Health (2014). Skolbarns Hälsovanor i Sverige 2013/14. Health Behaviors among School-Aged Children in Sweden 2013/14.

[9] Olweus D. (1993). Bullying at School: What We Know and What We Can Do.

[10] Finkelhor D., Turner H.A., Hamby S. (2012). Let’s prevent peer victimization, not just bullying. Child Abuse Negl..

[11] Modin B., Låftman S.B., Östberg V. (2015). Bullying in context: An analysis of psychosomatic complaints among adolescents in Stockholm. J. Sch. Violence.

[12] Modin B., Östberg V. (2009). School climate and psychosomatic health: A multilevel analysis. Sch. Eff. Sch. Improv..

[13] Meilstrup C., Ersbøll A.K., Nielsen L., Koushede V., Bendtsen P., Due P., Holstein B.E. (2015). Emotional symptoms among adolescents: Epidemiological analysis of individual-, classroom- and school-level factors. Eur. J. Public Health.

[14] Kalter F., Heath A.F., Hewstone M., Jonsson J.O., Kalmijn M., Kogan I., van Tubergen F. (2013). The Children of Immigrants Longitudinal Survey in Four European Countries (CILS4EU): Motivation, Aims, and Design.

[15] CILS4EU Children of Immigrants Longitudinal Survey in Four European Countries. http://cils4.eu/images/wave1_material/technical/za5353_technicalreport_wave1.pdf.

[16] Alm S., Låftman S.B. (2016). Future orientation climate in the school class: Relations to adolescent delinquency, heavy alcohol use, and internalizing problems. Child. Youth Serv. Rev..

[17] Mood C., Jonsson J.O., Låftman S.B. (2016). Immigrant integration and youth mental health in four European countries. Eur. Soc. Rev..

[18] Mood C., Jonsson J.O., Låftman S.B. (2017). The mental health advantage of immigrant-background youth: The role of family factors. J. Marriage Fam..

[19] Analitis F., Velderman M.K., Ravens-Sieberer U., Detmar S., Erhart M., Herdman M., Berra S., Alonso J., Rajmil L. (2009). European Kidscreen Group. Being bullied: Associated factors in children and adolescents 8 to 18 years old in 11 European countries. Pediatrics.

[20] Hjern A., Rajmil L., Bergström M., Berlin M., Gustafsson P.A., Modin B. (2013). Migrant density and well-being: A national school survey of 15-year-olds in Sweden. Eur. J. Public Health.

[21] Plenty S., Jonsson J.O. (2017). Social Exclusion among peers: The role of immigrant status and classroom immigrant density. J. Youth Adolesc..

[22] Låftman S.B., Modin B., Östberg V. (2013). Cyberbullying and subjective health. A large-scale study of students in Stockholm, Sweden. Child. Youth Serv. Rev..

[23] Tippettt N., Wolke D. (2014). Socioeconomic status and bullying: A meta-analysis. Am. J. Pub. Health.

[24] Hjalmarsson S. (2017). Poor Kids? Economic Resources and Adverse Peer Relations in a Nationally Representative Sample of Swedish Adolescents. J. Youth Adolesc..

[25] Reiss F. (2013). Socioeconomic inequalities and mental health problems in children and adolescents. A systematic review. Soc. Sci. Med..

[26] Ganzeboom H.B.G., de Graaf P., Treiman D.J. (1992). A Standard International Socio-Economic Index of Occupational Status. Soc. Sci. Res..

[27] Ganzeboom H.B.G. International Standard Classification of Occupations ISCO-08 with ISEI-08 Scores, Version July 27 2010. http://www.harryganzeboom.nl/isco08/isco08_with_isei.pdf.

[28] Chester K.L., Callaghan M., Cosma A., Donnelly P., Craig W., Walsh S., Molcho M. (2015). Cross-national time trends in bullying victimization in 33 countries among children aged 11, 13 and 15 from 2002 to 2010. Eur. J. Public Health.

